# Perturbations biologiques au cours du paludisme: à propos de trente cas

**DOI:** 10.11604/pamj.2017.26.174.9008

**Published:** 2017-03-27

**Authors:** Assya Khermach, Hanane Khalki, Lhoussine Louzi, Ali Zinebi, Karim Moudden, Mohamed Elbaaj

**Affiliations:** 1 Service de Parasitologie-Mycologie, Hôpital Militaire Moulay Ismail Meknès, Maroc; 2 Service de Médecine Interne, Hôpital Militaire Moulay Ismail Meknès, Maroc

**Keywords:** Paludisme, Plasmodium ovale, Plasmodium falciparum, cholestérol, thrombopénie, Malaria, Plasmodium ovale, Plasmodium falciparum, cholesterol, thrombopenia

## Abstract

L’infection palustre est une parasitose qui s’accompagne assez fréquemment de perturbations hématologiques et biochimiques. Quoique non spécifiques, ces signes biologiques peuvent être évocateurs du paludisme chez des patients ayant séjourné en zone d’endémie et présentant des signes cliniques de la maladie. Nous avons essayé à travers cette étude d’apprécier la fréquence de ces perturbations chez une série de cas avec paludisme d’importation. Nous avons analysé la NFS, le bilan lipidique, la CRP et la LDH chez une série de 30 cas de paludisme. La thrombopénie était présente chez 90% des cas (n=27) et l’anémie chez 23% des cas (n=7). La CRP était augmentée chez tous les cas. L’hypocholestérolémie était chez 80% des cas (n=24), l’hypoHDLémie chez 93,3% des cas (n=28), l’hypertriglycéridémie chez 36,6% (n=11) et la LDH était élevée chez 53,3% des cas (n=16). Ainsi, devant une suspicion d’infection palustre avec un examen direct négatif, la présence associée de ces perturbations biologiques augmenterait la probabilité diagnostique en faveur du paludisme.

Malaria infection is a parasitic disease often associated with hematological and biological disturbances. Although not specific, these biological signs may be suggestive of malaria in patients who stayed in endemic areas and showing clinical signs of the disease. Our study aimed to assess the frequency of these disturbances in a series of cases with imported malaria. We analyzed NFS, the lipid profile, CRP and LDH in a series of 30 cases of malaria. Thrombopenia was found in 90% of cases (n = 27) and anemia in 23% of cases (n = 7). CRP increased in all cases. Hypocholesterolemia was found in 80% of cases (n = 24), hypoHDLemia in 93.3% of cases (n = 28), hypertriglyceridemia in 36.6% (n = 11) and LDH increased in 53.3% of cases (n = 16). Thus, where there is suspicion of malaria infection with negative direct examination, the presence of both these biological disturbances would increase the diagnostic probability of malaria.

## Introduction

Le paludisme est l’infection parasitaire la plus fréquente dans le monde. Elle est transmise par un moustique du genre *Anopheles*. Quatre espèces principales sont en causes: *Plasmodium falciparum*, *P. ovale*, *P. vivax* et *P. malariae* [1]. Sur le plan mondial, c’est une priorité de santé publique vu qu’il est une des premières causes infectieuses de mortalité dans les zones d’endémie. En dehors de ces zones, on parle du paludisme d’importation. C’est une urgence diagnostique et thérapeutique surtout quand il s’agit de sa forme grave [2]. Les décès sont le plus souvent dus à un retard diagnostique [3]. L’examen microscopique du frottis sanguin coloré au May-Grünwald-Giemsa (MGG) associé à la goutte épaisse reste le Gold standard pour le diagnostic de cette pathologie. Cependant, il est examinateur dépendant, et dépend également de la qualité du frottis réalisé. De surcroit, dans les formes où la parasitémie est très basse, avec un contexte épidémiologique et clinique très évocateurs, le diagnostic reste très difficile. Le recours aux autres examens biologiques permet souvent de déceler des perturbations hématologiques et biochimiques en réponse à la phase aigüe de l’infection. Ainsi, tout élément permettant de contribuer au diagnostic doit être pris en compte pour évaluer la probabilité diagnostique [4]. L’objectif de notre étude était de mettre en évidence les perturbations biologiques observées pour une série de cas marocains avec paludisme d’importation.

## Méthodes

Il s’agit d’une étude rétrospective s’étalant sur une période de quatre ans allant du Janvier 2012 à Décembre 2015 et portant sur 30 cas paludéens, hospitalisés au service de médecine interne de l’hôpital militaire Moulay Ismail de Meknès. Le diagnostic du paludisme a été retenu par l’observation des hématozoaires du parasite sur la goutte épaisse et le frottis sanguin colorés au MGG. Nous avons analysé la numération formule sanguine, le bilan lipidique, la protéine C réactive (CRP) et la lactodéshydrogénase (LDH) qui ont été réalisés comme examens complémentaires à l’admission des malades. Nous avons utilisés le test khi-deux pour la comparaison des proportions des cas avec et sans perturbations biologiques. L’analyse a été faite sur Epi Info^®^ 7.

## Résultats

L’âge moyen des patients était de 32 ans, extrêmes: [20-49ans]. Tous nos malades étaient de sexe masculin. Ils ont tous séjourné en Afrique sub-saharienne : Côte d’Ivoire (21 cas), République Démocratique du Congo (9cas). La totalité des patients ont présenté une fièvre élevée. Le P. ovale était en cause chez 60% des cas (n=18) et le *P.falciparum* chez 40% des cas (n=12). L’anémie était présente dans 23,3% sans différence statistiquement significative entre le groupe infecté par le P. ovale et celui infecté par le *P. falciparum* (p= 79).

Le taux des globules blancs ainsi que celui des polynucléaires neutrophiles était normal chez la totalité des patients. Cependant, une lymphopénie a été observée chez 50% des cas (n=15) et une thrombopénie a été observée chez 90% des cas (n=27). La LDH a été augmentée chez 58, 3% (n=16). Nous n’avons pas observé de différence statistiquement significative pour ces trois derniers paramètres entre le groupe de patients infectés: p=0,70 ; p=0,71 et p=0,94 respectivement.

Les perturbations lipidiques les plus caractéristiques étaient une hypocholestérolémie avec un taux inférieur à 1,1g/l. Il n’y avait pas de différence statistiquement significative entre les patients infectés par le *P. ovale* et ceux infectés par le *P. falciparum* (p=0,30). Une hypoHDLémie a été retrouvée chez 93,3% des cas (n=28), et une hypertriglycéridémie dans 36,6% des cas (n=11) sans qu’il y ait aussi de différence entre les deux groupes de patients (p=0,65 ; p=0,94 respectivement). La CRP était augmentée chez la totalité de nos malades (Tableau 1).

**Tableau 1:** Perturbations biologiques observées chez nos cas au cours de la phase aigüe du paludisme

Perturbations Biologiques	*P. ovale*		*P.falciparum*		Total	
	Nombre de cas	%	Nombre de cas	%	Nombre de cas	%
Anémie (Hb< 12g/dl)	4	22,2	3	25	7	23,3
Thrombopénie (plaquettes<150.10^3^/μl)	16	88,9	11	91,6	27	90
Lymphopénie (lymphocytes<1000.10^3^/μl )	8	44,4	7	58,3	15	50
Hypocholestérolémie (CT<1,1g/l )	16	88,8	8	66,6	24	80
HypoHDLémie ( HDL<0,3g/l )	17	94,4	11	91,6	28	93,3
Hypertriglycéridémie (TG>1,7 g/l)	7	38,8	4	33,3	11	36,6
Elévation de la CRP (> 4,5mg/l)	18	100	12	100	30	100
Elévation de la LDH (> 230UI/l)	9	50	7	58,3	16	53,3

## Discussion

Le diagnostic du paludisme repose habituellement sur l’association des données épidémiologiques (retour d’une zone endémique), des signes cliniques en faveur où la fièvre constitue un argument compatible mais non exclusif et la démonstration microbiologique de la présence du parasite. Un certain nombre de perturbations biologiques peuvent être prises en compte, principalement : la thrombopénie, la lymphopénie, l’hypocholestérolémie majeure avec hypoHDLémie, l’élévation de la CRP et de la LDH [4].

L’anémie est une anomalie commune chez les patients paludéens. Son mécanisme physiopathologique pourrait être en rapport soit avec une augmentation de l’hémolyse intravasculaire des globules rouges parasités ou avec une diminution de leur production [5]. Nos résultats concernant l’anémie se rapprochent de ceux de la série de Winters et al [6] et Richards et al [7]. Cependant, l’anémie était plus marquée dans la série de Rodrigues et al [8] (Tableau 2).

**Tableau 2:** Tableau comparatif de l’anémie observée dans notre série et celle observée dans trois études

Auteurs	Années et lieux d’étude	Nombre de cas avec anémie	Pourcentage des cas avec anémie %
Winters et al.[6] (n=86)	New York (Etats unis) 1968-1975	22	26
Richards et al.[7] (n=89)	London (Etats unis) 1994	13	15
Rodrigues et al.[8] (n=71)	Porto Velho (Brazil) 2013	33	46,5
Notre série (n=30)	Meknès (Maroc) 2012-2015	7	23,3

La thrombopénie a été fréquemment associée à l’accès palustre mais elle n’est pas constante [9]. Les mécanismes les plus couramment incriminés sont la destruction et la consommation des plaquettes au niveau splénique. En effet, le complexe immun fait de l’antigène malarique et les plaquettes fragilisées est séquestré par les macrophages et mené jusqu’à la rate où il est détruit [5]. Cependant, les troubles hémorragiques secondaires à la thrombopénie au cours du paludisme sont très rares [10]. La thrombopénie est une perturbation fréquente au cours du paludisme. Elle peut être utilisée comme un marqueur sensible mais non spécifique pour une infection active à *Plasmodium* [11]. En effet, d’après Erhart et al, les patients fébriles avec un taux de plaquettes <150G/l sont 12 à 15 fois plus susceptibles d’avoir un accès palustre que le groupe témoin [5]. Concernant la thrombopénie, les résultats de notre série (90%) corroborent avec ceux de la série de Al Fandari et al. (85,6%) et ceux de la série de Badiaga et al. (82%) [12, 13] (Tableau 3). Une leucopénie modérée a été fréquemment rapportée dans le paludisme. Elle peut être expliquée par l’augmentation du pool marginal et la réduction du pool circulant [4]. La variation du taux des leucocytes est dynamique. En effet, l’amélioration clinique s’accompagne d’un retour à la normale de ce taux. D’où son utilité pour la surveillance clinique [8].

**Tableau 3:** Tableau comparatif de la thrombopénie observée dans notre série avec celle observée dans six études

Etudes	lieux et années de l’étude	Nombre de cas avec thrombopénie	Pourcentage de cas avec thrombopénie (plaquettes<150G/L)
Richards et al. [7] (n=89)	London (Etats unis) 1994	60	67%
Al fandari et al. [12] (n=111)	Tourcoing (France) 1989-1994	95	85,6%
Badiaga.S et al. [13] (n=129)	Marseille (France) 1994-1997	106	82%
Mabiala-Babela et al. [14] (n=269)	Brazzaville (Congo) 2012-2013	170	63,2%
Chagnon et al. [16] (n=64)	Toulon (France) 1985-1991	45	70%
Notre etude (n=30)	Meknès (Maroc) 2012-2015	27	90%

Nous n’avons trouvé aucun cas avec une leucopénie, ce qui pourrait être considéré proche des résultats trouvés par Mabiela-Babela et al. [14] qui n’ont révélé cette anomalie que chez 2,23%. Cependant, Badiaga et al. [13] ont mis en évidence cette baisse des leucocytes chez 47% (Tableau 4). En plus d’un comptage des globules blancs lors du paludisme, leur aspect morphologique peut être également affecté. La présence du pigment malarique dans les leucocytes est très évocatrice du paludisme [3].

**Tableau 4:** Tableau comparatif de la leucopénie observée dans notre série avec celle observée dans trois études

Etudes	Lieux et années de l’étude	Nombre de patients ayant une leucopénie	Pourcentage des patients ayant une leucopénie %
Winters et al. [6] (n=86)	New York (Etats unis) 1968-1975	9	10
Badiaga et al. [13] (n=129)	Marseille (France) 1994-1997	60	47
Mabiala-Babela. [14] (n= 269)	Brazzaville (Congo) 2012-2013	6	2,23
Notre série (n=30)	Meknès (Maroc) 2012-2015	0	0

Parmi les leucocytes, les lymphocytes sont les plus touchés par ces modifications hématologiques. Les mécanismes physiopathologiques responsables de la lymphopénie sont complexes ; plusieurs hypothèses ont été émises. Elle serait due à la séquestration des lymphocytes dans les tissus ou les organes actifs tels que la rate. Un rétrocontrôle négatif suite à une hyperstimulation immunitaire ou dans le cadre d’un échappement immunitaire du parasite, une réduction de la durée de vie des lymphocytes suite de l’expression des facteurs apoptotiques Fas et Fasl a été observée dans un modèle animal [15].

Dans notre série, la lymphopénie était présente dans la moitié des cas. Nos résultats étaient concordants avec ceux de la série de Chagnon et al. et Richards et al [7, 16] avec une lymphopénie observée dans 44 et 63% respectivement (Tableau 5). De nombreux auteurs ont démontré une modification des taux lipidiques au cours d’un état fébrile ou septique. En effet, l’association d’une hypocholestérolémie et d’une hypertriglycéridémie est la plus fréquemment retrouvée pendant la phase aigüe d’une infection. Ces modifications s’accentuent dans les trois premiers jours de l’infection [17].

**Tableau 5:** Tableau comparatif de la lymphopénie observée dans notre série avec celle observée dans deux études

Etude	Lieux et années de l’étude	Nombre de patients avec une lymphopénie	Pourcentage des patients avec une lymphopénie
Richards et al. [7] (n=89)	London (Etats unis) 1994	52	63
Chagnon et al. [16] (n=64)	Toulon (France) 1985-1991	28	44
Notre série (n=30)	Meknès (Maroc) 2012-2015	15	50

Les résultats de notre série se rapprochent de ceux de Chukuocka et al. [18]. Cependant, dans la série de Badiaga et al. et Chagnon et al., seulement 40% et 45% ont présenté une hypocholestérolémie [13, 16] (Tableau 6).

**Tableau 6:** Tableau comparatif de l’hypocholestérolémie observée dans notre série avec celle observée dans trois études

Etude	Lieux et années de l’étude	Nombre de cas avec une hypocholestérolémie	Pourcentage des patients avec une hypocholestérolémie%
Badiaga et al. [13] n=53/129)	Marseille (France) 1994-1997	21	40
Chagnon et al. [16] (n=64)	Toulon (France) 1985-1991	29	45
Chukuocha et al.[18]	Owerri(Nigeria) 2011	46	84
Notre série (n=30)	Meknès (Maroc) 2012-2015	24	80

Les modifications du bilan lipidique touchent surtout la fraction HDLc. Lambrecht et al, ont montré l’absence de HDLc chez six patients infectés par P. vivax [19]. Cette hypoHDLémie peut être expliquée par une inhibition de la lécithine-cholestérol acyl transférase et de la lipoprotéine lipase qui participent à l’élaboration de l’HDLc par le parasite ou ses produits [4]. Dans la série d’Al-Omar et al., les patients infectés par le *P. falciparum* avaient un taux de cholestérol significativement bas par rapport au groupe témoin [11]. Ils ont constaté également une corrélation inverse entre la parasitémie et le taux de cholestérol. Elle était considérée comme un indicateur spécifique (98%) de l’infection par le *Plasmodium* [13]. Cependant, Faucher et al, dans leur série de 112 patients ont démontré pour la première fois qu’une parasitémie faible à *P.falciparum* a modifié le profil lipidique chez ces patients [20].

L’hypoHDLémie a été présente dans notre série dans 93,3% des cas. Baptisa et al. ont démontré dans leur étude, une diminution significative des taux de l’HDL cholestérol chez les patients paludéens [21]. Cette diminution peut être expliquée par l’inhibition de la lécithine-cholestérol estérifié par le *Plasmodium* ou ses produits qui entrainent une réduction du transport du cholestérol estérifié au foie, elle peut être due également à l’inhibition de la lipoprotéine lipase qui entre dans la synthèse du HDL cholestérol [4]. Dans notre série, on note une hypertriglycéridémie dans 36,6% de nos malades. Sur ce point, Chagnon et al. sont d’accord [16]. Cette hypertriglycéridémie peut être expliquée par la réduction de l’activité de la lipoprotéine lipase due à l’épuration des TG du compartiment plasmatique [21].

La protéine C réactive, un marqueur d’inflammation le plus utilisé en clinique, a été significativement élevé dans plusieurs séries des cas de paludisme [22, 23]. Elle est utilisée également comme un indicateur discriminant des différentes formes cliniques du paludisme. En effet, Kamgaing et al. ont trouvé une différence significative entre les enfants avec un paludisme grave de ceux ayant un paludisme simple [23]. Quant à notre série, tous nos malades avaient un taux de CRP élevé avec une moyenne de 72 mg/l.

La LDH était élevée dans 53,3% des cas. Dans le même sens, Winters et al. ont trouvé dans leur série une élévation de la LDH dans 83% (n=63/76) [6]. L’augmentation de ce paramètre pourrait être probablement due à l’hémolyse intravasculaire [4].

## Conclusion

Quoique non spécifique, la perturbation regroupée de ces paramètres, associée à des données cliniques avec notion de séjour en zone d’endémie, augmente la probabilité diagnostique en faveur du paludisme malgré une recherche négative du parasite, ou encore nous incite à réexaminer d’une manière plus attentive et sur une durée plus longue la goutte épaisse et le frottis sanguin afin de déceler d’éventuels hématozoaires du parasite.

### Etat des connaissances actuelles sur le sujet

- Le paludisme est une infection parasitaire la plus répandue dans le monde;
- Le frottis sanguin et la goutte épaisse restent le Gold standard pour le diagnostic du paludisme;
- C’est une urgence diagnostique et thérapeutique surtout quand il s’agit de sa forme grave.

### Contribution de notre étude à la connaissance

- Dans les formes à parasitémie basse, le diagnostic du paludisme restent très difficile;
- La présence d’un bilan hématologique et biochimique perturbé dans un contexte épidémiologique et clinique évocateur, peut contribuer au diagnostic en faveur du paludisme;
- L’anémie, la thrombopénie, l’hypocholestérolémie, l’hypoHDLémie, l’augmentation de la CRP et de la LDH sont souvent retrouvés en cas du paludisme et peut aider au diagnostic en cas de parasitémie basse.
